# Successful IVC thrombus extraction with the AngioVac device following five vessel coronary artery bypass graft: a case report

**DOI:** 10.1186/s13019-021-01605-9

**Published:** 2021-08-09

**Authors:** Steven Neubauer, Gianmarino Gianfrate, Lucas Henn

**Affiliations:** Department of General Surgery, Mercy Health St. Elizabeth Youngstown Hospital, 1044 Belmont Ave, Youngstown, OH USA

**Keywords:** IVC filter thrombosis, CABG, Thrombectomy, AngioVac system, Case report

## Abstract

**Background:**

Inferior vena cava thrombosis is cited to be a complication of inferior vena cava filter placement and post coronary artery bypass surgery. Often only mild symptoms arise from these thrombi; however, due to the chronic nature of some thrombi and the recanalization process, more serious complications can arise. Although anticoagulation remains the gold standard of treatment, some patients are unable to be anticoagulated. In this case, we present a 65-year-old male who underwent IVC filter placement and open-heart surgery who later developed extensive femoral and iliocaval thrombosis leading to right heart failure, which required thrombus extraction with an AngioVac suction device.

**Case presentation:**

We present a 65-year-old male who presented with bilateral pulmonary emboli with extensive right lower extremity deep vein thrombosis. Upon investigation he had ischemic heart disease and underwent a five-vessel coronary artery bypass for which he had an IVC filter placed preoperatively. On post operative day 3 to 4, he was decompensated and was diagnosed with an IVC thrombus. He progressed to right heart failure and worsening cardiogenic shock despite therapeutic anticoagulation and was taken for a suction thrombectomy using the AngioVac (AngioDynamics, Latham, NY) aspiration thrombectomy device. The thrombectomy was successful and he was able to recover and was discharged from the hospital.

**Conclusion:**

Despite being a rare complication, IVC thrombosis can have detrimental effects. This case is an example of how IVC thrombus in the post-operative setting can lead to mortality. The gold standard is therapeutic anticoagulation but despite that, this patient continued to have worsening cardiogenic shock. Other therapies have been described but because of its rarity, they are only described in case reports. This case shows that the AngioVac device is a successful treatment option for IVC thrombus and can have the possibility of future use.

## Background

Inferior vena cava (IVC) thrombosis is a rare but known complication of both IVC filter placement and open coronary artery bypass. Multiple recent research reviews support this notion, showing an increase incidence of IVC thrombosis with patients undergoing open coronary bypass when compared to other general surgeries (2.5%, 0.4%, p < 0.05) [1]. Along  with coronary artery bypass, Nazzal et al., reiterated the complications of IVC filters, one of which is thrombosis [2]. Although both are rare causes of thrombosis, the usual symptoms are ambiguous and can vary based on acuity [2]. The most common complaints are bilateral lower extremity swelling, back pain, and cramping [2, 3]. Seldomly these thrombi can be extensive enough to lead to severe symptoms that result in anuria, right heart failure, pulmonary emboli and cardiac arrest [3, 4]. Treatment is typically anticoagulation and, in the case of filter placement, the removal of the IVC filter [2, 3]. Over the last couple of years, there has been a multitude of reports of patients needing operative intervention due to systemic symptoms from an IVC thrombus [1–4]. We present a case of a 65-year-old male who developed an IVC thrombus post operatively from a coronary artery bypass and IVC filter placement causing cardiogenic shock which required intervention with the Angiovac thrombectomy device.

## Case report

A 65-year-old male with past medical history of diabetes, hypertension, hyperlipidemia, obstructive sleep apnea (OSA), obesity, previous dermatofibrosarcoma protuberans presented to the emergency department with acute onset shortness of breathing and chest pain. He presented during the COVID-19 pandemic when he tested negative for the disease. He was diagnosed with bilateral pulmonary emboli and extensive DVT in his right leg, which was initially treated with therapeutic anticoagulation with a heparin infusion. He had an echocardiogram (echo) to rule out right heart strain that showed newly diagnosed ischemic cardiomyopathy with a reduced ejection fraction estimated between 30 and 35% and global hypokinesis. He was transferred to a higher level of care for a cardiac catherization which revealed severe multivessel coronary disease with global hypokinesis. Cardiac surgery was consulted and recommended 5-vessel bypass of his left anterior descending artery, obtuse marginal, posterior descending artery, and right posterolateral artery with thromboembolectomy.

Vascular surgery was consulted for inferior vena cava (IVC) filter placement due to his DVT and need to be off anticoagulation for cardiac surgery. He had the IVC filter (Cook medical, Bloomington, IN) placed two days prior to his cardiac bypass by access through right common femoral vein and placed at lumbar vertebra 3. He then underwent a 5-vessel coronary artery bypass (CABG) using internal mammary artery, left radial artery, and right saphenous vein grafts. He also had thromboembolectomy of bilateral pulmonary veins. He was transferred to the stepdown unit on post op day 1 and was progressing appropriately postoperatively. On post op day 3, he had a syncopal event while ambulating and felt a pop in his right groin region and started having progressive swelling and loss of motor and sensation of his right lower extremity. He had tight calf and thigh compartments on exam and decreased doppler pulse exam from previous. Of note on his morning laboratory values he had a decrease in his hemoglobin and increase in his creatinine. He went for emergent fasciotomy and evacuation of large amount of hematoma from his calf and thigh.

Post procedure he remained critical and hypotensive with pressor support with epinephrine and vasopressin. He had a repeat echo which did not show any abnormality or concern for graft failure. He proceeded to go into multisystem organ failure. He required a temporary dialysis catheter placement. The catheter continued to have clotting issues while in the femoral veins. On post op day 4 from his CABG, he continued to have progressive mottling and severe lower extremity edema with worsening shock state. He was started on Argatroban through his femoral central venous catheter (CVC) for concern for IVC thrombus based on clinical exam.

**Fig. 1:**
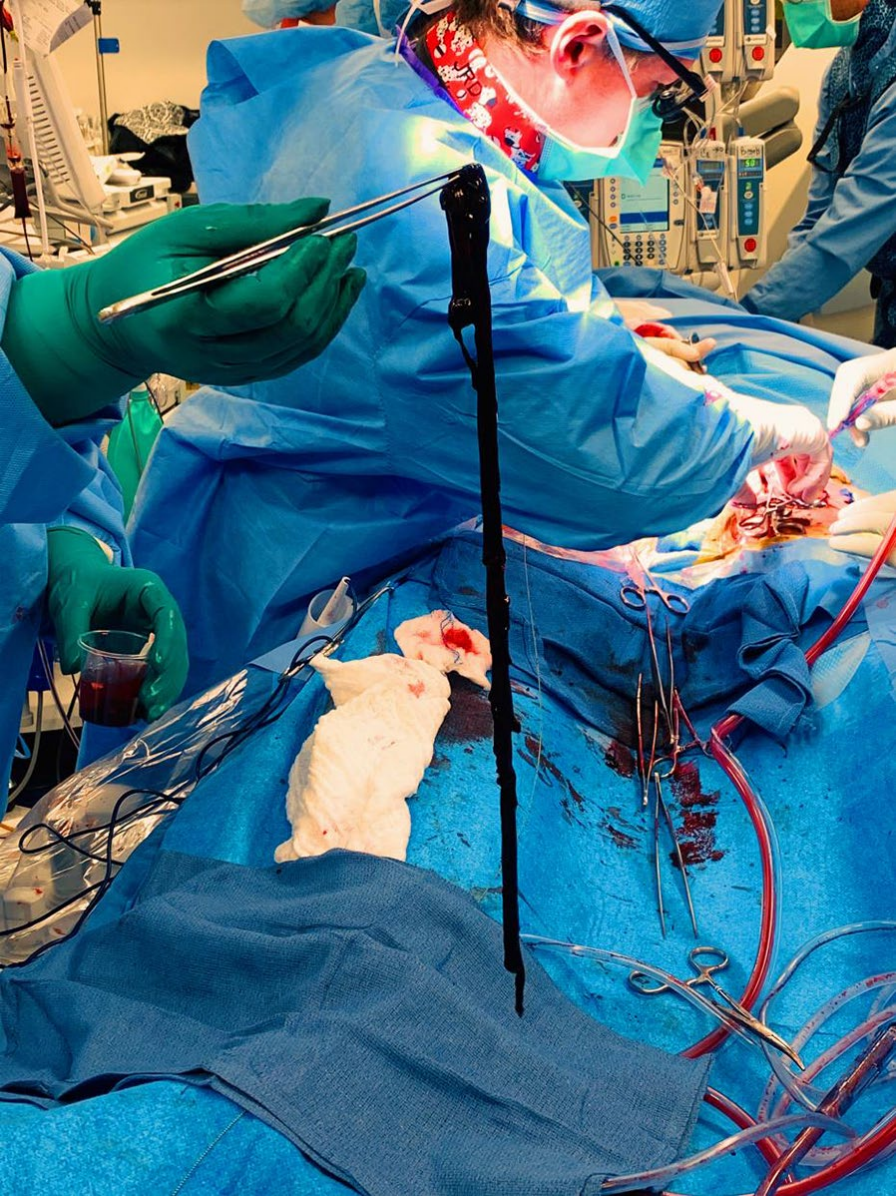
Thrombus removed for the right lower extremity during the mechanical thrombectomy

**Fig. 2:**
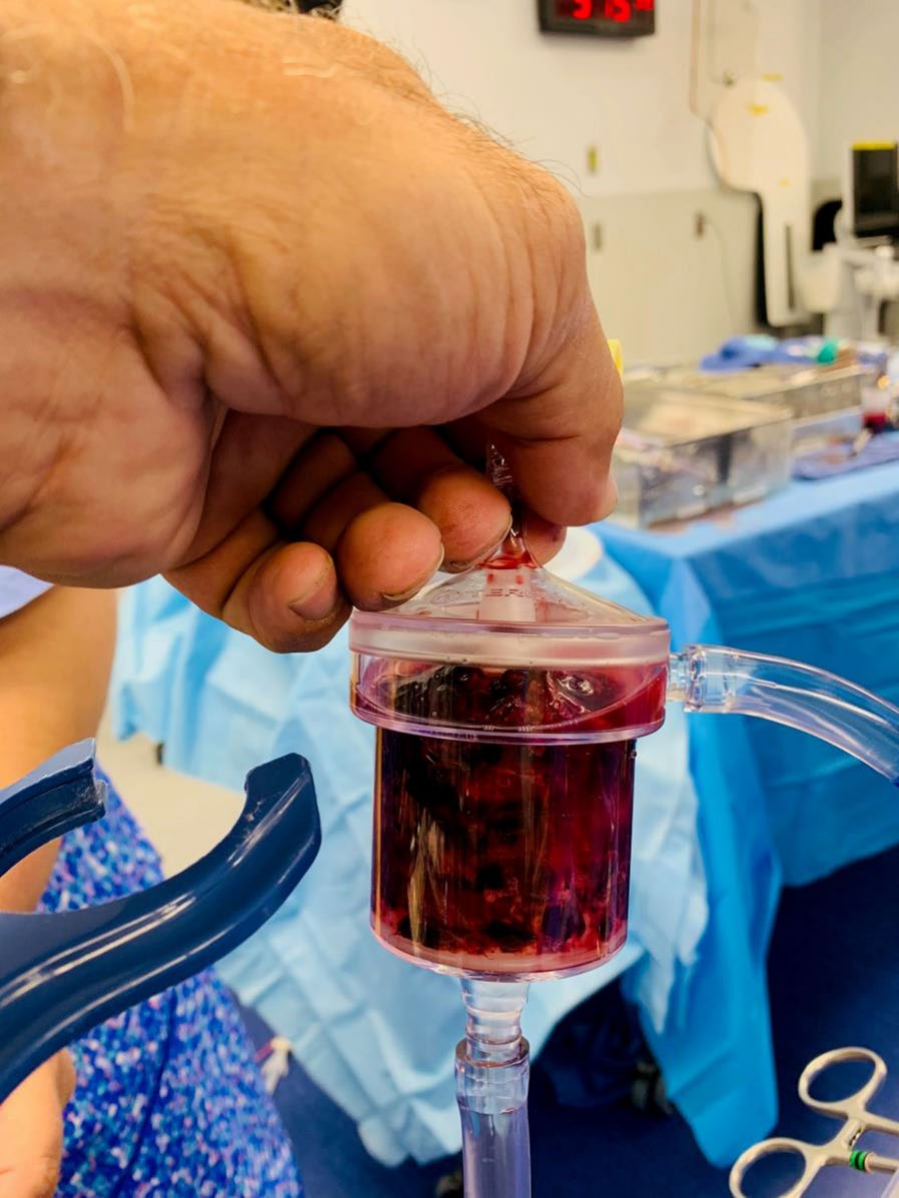
AngioVac canister after suction thrombectomy was performed. This was the first on two canisters filled with thrombus

**Fig. 3:**
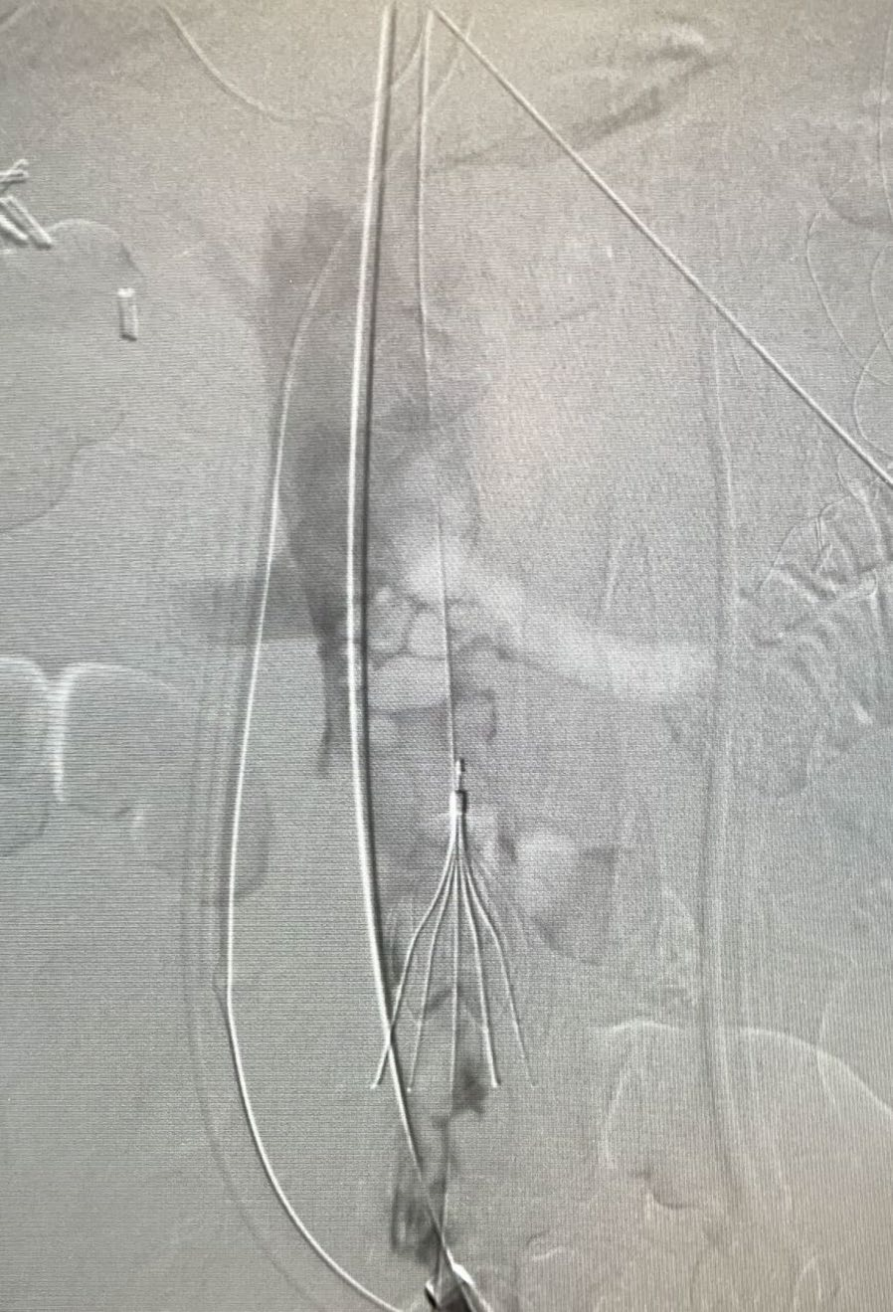
Venogram of IVC with IVC filter in place prior to suction thrombectomy with AngioVac device. This shows extensive clot burden at the level of the IVC filter and extending superior into the renal veins

**Fig. 4:**
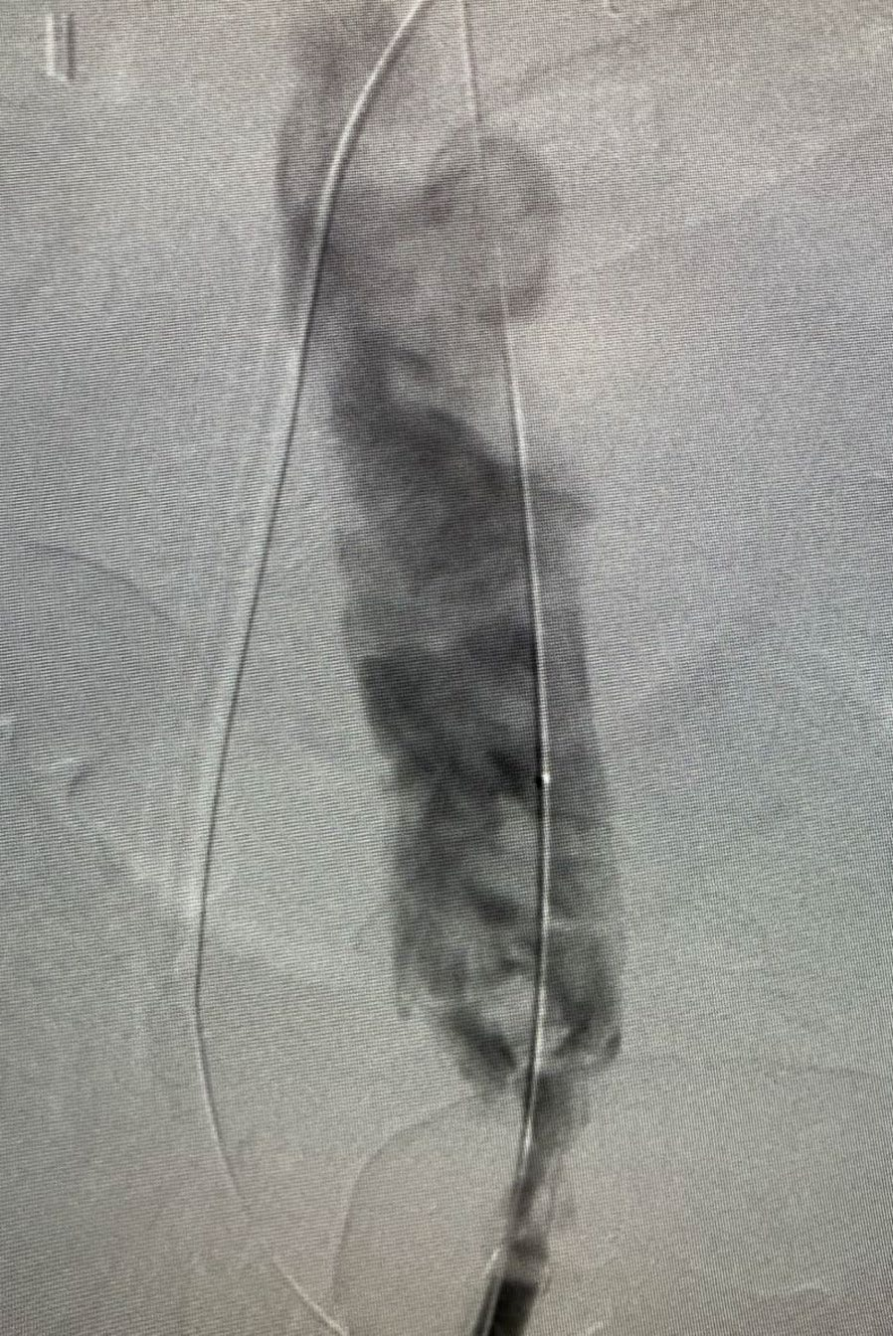
Venogram post suction thrombectomy with the AngioVac device

After discussion with cardiac team and vascular team, he was taken for venogram and thrombectomy due to his continued decline despite being therapeutic on anticoagulation. Bilateral groin incisions were used and thrombectomies of bilateral femoral veins proximal and distal were performed with removal of large amount of clot (Fig. 1). A venogram was performed which showed clot extending to IVC filter and into bilateral renal veins (Fig. 2). The right internal jugular vein was accessed and the IVC filter was repositioned suprarenal. The right internal jugular vein was then used for the Angiovac return sheath, while the right groin was used for the Angiovac suction sheath. Under transesophageal echo (TEE), the Angiovac system was used for suction thrombectomy of the entire IVC and removal of clot from the right atrium (Fig. 3). A post thrombectomy venogram was performed as well (Fig. 4). He was continued on Argatroban post operatively.

Throughout the post-operative course, he was able to be weaned from his presser requirement, required a tracheostomy and gastrotomy tube, and required a tunneled catheter for his acute renal failure. He had a repeat echo which showed an ejection fraction of 40% without any wall abnormality. His anticoagulation was changed to coumadin. His hypercoagulable workup including JAK2, prothrombin gene mutation, Factor 5 Leiden, anticardiolipin antibody IgG and IgM, beta-2 glycoprotein IgG and IgM, Homocystine, and Protein S were negative for any hypercoagulable disorder. He was discharged to a long-term care facility and then proceeded to go to acute rehab facility. He did well and progressed well. He had his tracheostomy removed along with his gastrotomy tube. His renal function did recover and ultimately had his tunneled dialysis catheter removed and his IVC filter removed about 6 months after his operation. His last follow up with cardiac surgery was 3 months after his operation and has had no cardiac issues since. Today he is ambulating well but still needs help with some daily activities.

## Discussion

Inferior Vena Cava thrombosis is a possible complication in many critically ill and surgical patients. Although rare, it can be associated with potentially fatal complications [1–4]. An IVC thrombosis is an under-recognized complication that can lead to severe mortality and morbidity. It has been estimated that upwards of 4% of patients with lower extremity DVT also have a corresponding IVC thrombosis [4]. With the mortality rate increased two-fold with IVC thrombosis when compared to lower limb DVT, IVC thrombosis is a major complication [3, 4]. Other causes are coronary artery bypass and inferior vena cava filter placement. Patients who undergo cardiac surgery have significantly impaired functional cardiorespiratory reserve in the post-operative period. It is this impairment that removes these individuals’ ability to compensate, even for a short period of time. A small deterioration in their cardiopulmonary system, i.e. a venous thrombosis, can have systemic effects. Although rare, it is well-document that there is an increase in the development of venous thrombosis in post cardiac patients [1]. In a large study comparing the incidence of IVC thrombosis, there was a significant increase in the development of a thrombosis in those that underwent coronary artery bypass when compared to other large general surgery operations (2.5% compared to 0.4%, p < 0.05) [1].

Although open heart surgery plays a role in developing venous thrombosis of the great vessel, IVC filter placement can also play a significant role in development and propagation of a thrombosis [2–4]. First line therapy for venous thrombosis and prevention of pulmonary embolism remains anticoagulation. However, often critical ill patients with recent gastrointestinal bleeding or hemorrhagic traumatic brain injuries cannot be anticoagulated due to a major risk of hemorrhage. Over the last 40 plus years many second-line treatment options have increase in popularity [4]. One of these modalities is placement of an IVC filter. First used in 1967, with the first successful percutaneous placement in 1984, the reverse cone shaped device has proven to be effective in prevention of PEs [2, 3]. Although they help to prevent the development of PEs, many of these patients underlying DVT remained with even some developing propagation of the DVT [2, 3]. One major complication that can arises in patients with IVC filter is an IVC thrombosis. Nazzal et al. showed that 4.75% of patient that received an IVC filter at their institution between the years of 2002 to 2006 went on to develop an IVC thrombosis [2]. There are many ideas to why an IVC filter leads to the development of a thrombosis [2]. Two of the most accepted methods are expansion of pre-existing clot or endovenous injury [2–4]. A pre-existing ilio-femoral DVT can easily propagate into the IVC, especially if this is the only means for PE prophylaxis [3, 4]. As the thrombosis gets larger, it could eventually involve the renal veins leading to a renal vein thrombosis. This can manifest as anuria, elevation in creatinine and ultimately acute kidney failure, as in our patient [5]. IVC thrombosis secondary to endovenous intervention, such as IVC filter placement, could also lead to a thrombosis in situ. This could be due to direct venous injury at the puncture site in the femoral vein or from the filter within the IVC [6, 7]. The IVC filter can act as a foreign body leading to coagulation activation and recruitment [6]. Although IVC thrombosis secondary to IVC filter is an issue, most of the time the thrombosis never causes a full occlusion of the vein. Over time it has been shown that chronic occlusion usually recanalize [7].

IVC thrombus, although rare, are prevalent in the United States due to presumed overutilization of IVC filter and low rates of retrieval of filters [4]. Other complications of an IVC thrombus are post thrombotic syndrome, claudication, pulmonary embolism, renal vein thrombus and phlegmasia cerula dolens [5, 6, 8]. Some patients, as in this case, progress becoming unstable and are in need of therapeutic intervention. One of the most common modalities of therapeutic intervention with extensive thrombi is the AngioVac system. AngioVac system is used to remove undesirable intravascular material within the right ventricle, Superior Vena Cava (SVC), Inferior Vena Cava (IVC) and iliofemoral vessels [8]. The AngioVac system acts as an extracorporeal bypass circuit which creates a one-way suction flow to extract the unwanted material out of the vessel [8]. The most common reason an AngioVac system is used is when there is the presence of a right heart thrombus [8, 9]. Varies sources have shown complete or partial success, with roughly an 80–90% success rate, when used in this situation [9]. Although this device has high success rates, its use it not for every patient. In the setting of chronic thrombi, the AngioVac system has been shown to have lower rates of success [8–10]. This is most likely secondary to the adherences that chronic thrombi have to the naïve vessel wall, making it difficult to dislodge [9, 10]. This system also has lower success rates when used in the setting of pulmonary emboli. This has been shown in multiple studies and is most likely due to the difficulties of accessing the pulmonary artery with the cannula [9–11]. Overall the AngioVac system seems to high success in patients who develop an acute extensive thrombus and are both poor candidates for extensive surgery and anticoagulation [8–11].

Specifically, to this case, the patient had 3 factors increasing their risk of an IVC thrombus; recent cardiac surgery, extensive DVT, and presence of IVC filter. Although this patient had multiple factors that led to the extensive propagation of his thrombosis, we believe the IVC filter may have played the greatest role in its development. The patient developed acute kidney failure secondary to the native thrombosis that formed near the IVC filter. This was one the first post-operative findings that this patient developed. Due to the extensive thrombosis at this location, outflow was most likely obstructed leading to acute renal failure and symptoms like phlegmasia cerula dolens. This obstruction required a fasciotomy, but ultimately progressed to cardiogenic shock likely due to the reduced venous return and decreased preload in the setting of recent cardiac bypass. While not having an absolute contraindication to anticoagulation, this patient required intervention due to their continued instability. Current studies show that suction thrombectomy can be successful, but most are case series or case studies [8–10]. Suction thrombectomy using the AngioVac device can be an essential tool in certain patients with an IVC thrombus.

## Data Availability

All data generated or analyzed during this study are included in this published article and its supplementary information.

## Abbreviations

IVC: Inferior vena cava
TEE: Transesophageal echo
CABG: Coronary artery bypass graft
PE: Pulmonary emboli
DVT: Deep vein thrombosis
Echo: Echocardiogram

## References

[1] Saranteas T, Kostopanag G, Tzoufi M, Drachtidi K, Knox GM, Panou F (2013). Incidence of inferior vena cava thrombosis detected by transthoracic echocardiography in the immediate postoperative period after adult cardiac and general surgery. Anaesth Intensive Care.

[2] Nazzal M, Chan E, Nazzal M (2010). Complications related to inferior vena cava filters: a single-center experience. Ann Vasc Surg.

[3] McAree BJ, O’Donnell ME, Fitzmaurice GJ, Reid JA, Spence RAJ, Lee B (2013). Inferior vena cava thrombosis: a review of current practice. Vasc Med.

[4] Alkhouli M, Morad M, Narins C (2016). Inferior vena cava thrombosis. JACC Cardiovasc Intervent.

[5] Janvier AL, Hamdan H, Malas M (2010). Bilateral renal vein thrombosis and subsequent acute renal failure due to IVC filter migration and thrombosis. Clin Nephrol.

[6] Georgiou NA, Katz DS, Ganson G, Eng K, Hon M (2015). Erratum to: CT of inferior vena cava filters: normal presentations and potential complications. Emerg Radiol.

[7] Poon WL, Luk SH, Yam KY, Lee ACW (2002). Mechanical thrombectomy in inferior vena cava thrombosis after caval filter placement: a report of three cases. Cardiovasc Intervent Radiol.

[8] Bjarnason H, Behrens G (2015). Venous thromboembolic disease: the use of the aspiration thrombectomy device AngioVac. Semin Interv Radiol.

[9] D'Ayala M, Worku B, Gulkarov I, Sista A, Horowitz J, Salemi A (2017). Factors associated with successful thrombus extraction with the AngioVac Device: an institutional experience. Ann Vasc Surg.

[10] Francis F, Salerno G. Percutaneously inserted AngioVac suction thrombectomy for the treatment of filter-related Iliocaval thrombosis. Vasc Dis Manag. 2015;12(3).

[11] Moriarty JM, Al-Hakim R, Bansal A, Park JK (2016). Removal of caval and right atrial thrombi and masses using the AngioVac device: initial operative experience. J Vasc Interv Radiol.

